# Response to: Commentary: Sex Differences in the Peripheral Immune System in Patients with Depression

**DOI:** 10.3389/fpsyt.2017.00231

**Published:** 2017-11-10

**Authors:** Badari Birur, Richard C. Shelton, Ellen M. Amrock, Li Li

**Affiliations:** ^1^Department of Psychiatry and Behavioral Neurobiology, University of Alabama at Birmingham, Birmingham, AL, United States; ^2^Birmingham VA Medical Center (VHA), Birmingham, AL, United States

**Keywords:** sex, depression, inflammation, stress, interleukin-6, hypothalamic–pituitary–adrenal axis

We would like to thank Breidenstein and colleagues for writing a commentary on our study titled “Sex Differences in the Peripheral Immune System in Patients with Depression” and appreciate the positive comments (1). We agree that even though our study points toward women showing higher levels of inflammation during depression, it is not possible to draw inferences about causality from our results due to the cross-sectional nature of the study. Therefore, a longitudinal study is warranted to determine the causal relationship between inflammation and major depressive disorder (MDD) (2).

With regard to interleukin-6 (IL-6), Breidenstein et al. pointed out that we did not identify elevated levels of IL-6 in depressed women compared with controls after controlling for body mass index nor a positive correlation between IL-6 and depression severity. However, our study did find elevated IL-6 in depressed females when compared with depressed males after controlling for body mass index (2). To further support this, one of our previous studies found higher circulating levels of IL-6 in MDD, which was largely explained by obesity (3). Although numerous correlative studies have found elevated IL-6 in depressed patients relative to controls, a recent meta-analysis using longitudinal studies showed that the weighted-mean effect size for IL-6 was relatively small (unadjusted *r* = 0.045, *p* = 0.007; adjusted *r* = 0.097, *p* = 0.06) (4). In addition, elevated circulating IL-6 has been reported in several animal models of depression in which chronic mild stress or learned helplessness was applied to establish depressive-like symptoms (5). Taken together, studies in humans and animal models have provided strong evidence that inflammation is altered and IL-6 may be elevated in a subset of depressed patients or animals that are exposed to stress. However, the role of elevated IL-6 levels in depressed patients may need to be interpreted in a sex-specific manner as well as to include other confounding factors such as obesity. Thus, more rigorous and longitudinal studies in humans are needed to establish the sex–IL-6 relationship in depressed patients.

Breidenstein et al. raise an important issue regarding the relationship between gonadal hormones and inflammation that referred to a paper focusing on an immune system-related disease, i.e., rheumatoid arthritis, instead of the observations in depression (6). Indeed, recent studies indicate that the relationship between gonadal hormones and cytokines is complicated and their relationship varies from disease to disease. For example, several clinical and experimental studies have showed a gender dimorphism of the immune and organ responsiveness in the susceptibility to and morbidity from shock, trauma, and sepsis. Specifically, studies indicate that androgens are responsible for the immunodepression after trauma hemorrhage in males. By contrast, female sex steroids seem to exhibit immunoprotective properties after trauma (7). Furthermore, postmenopausal women have higher basal levels of IL-6 and a larger IL-6 stress response than age-matched men in response to acute stress (8). Although gonadal hormones are not in the scope of our current study, we would like to include gonadal hormone measurements in future studies to explore its complicated roles in depression and to determine if those effects are sex and age dependent.

Another issue raised by Breidenstein et al. is the role of psychosocial stress as a possible mediator in the relationship between inflammation and MDD, as well as the hypothalamic–pituitary–adrenal (HPA) axis in the pathogenesis of MDD. Two other studies from our group have suggested that childhood maltreatment is positively correlated with elevated inflammatory markers (9, 10). Our observations were consistent with Mondelli et al.’s reports (11) that psychosocial stress was one of the main factors determining neuroinflammation. Stress is a major risk factor for depression and the HPA axis mediates stress response. Altered HPA axis activity in depressed patients has been associated with both childhood maltreatment and acute stress responses. One of our prior studies suggests that individuals with childhood maltreatment showed suppressed HPA axis activity measured by cortisol awakening curve; this effect was independent of depression (9). Our observations were supported by other research groups who found early life adverse experiences were associated with decreased salivary cortisol responses to awakening (12). Both Holsboer et al. and Gold et al. reported that depressed women with and without childhood trauma exhibited blunted adrenocorticotropic hormone response to corticotropin-releasing factor (13, 14). By contrast, Heim et al. reported that women with a history of childhood abuse with and without current major depression exhibited increased adrenocorticotropic hormone but normal cortisol responses to stress compared with controls (15). Taken together, an interconnected relationship between inflammation, HPA axis, and depression might be present, which definitely warrants further investigation in humans and in animal models.

To summarize, our study demonstrated that inflammation is related to depression; however, the association is sex specific. Understanding the influence of inflammation on women’s mental health may help enhance our understanding of the sex differences in depression as well as help clinicians choose effective antidepressants in the future. More longitudinal studies are needed to answer the causal relationship between inflammation and MDD. Currently, more studies are ongoing in our department to understand the link between sex, depression, inflammation, and the HPA axis. We thank Dr. Breidenstein and group for the thoughtful comments, which focus our attention to be careful as we describe and interpret our outcome measures, and helpful to design future studies.

## Author Contributions

BB and LL wrote, edited, and approved the submission. RS and EA edited and approved the submission.

## Conflict of Interest Statement

The authors declare that the research was conducted in the absence of any commercial or financial relationships that could be construed as a potential conflict of interest.

## References

[1] Breidenstein J Przyborowski C Walther A Commentary: sex differences in the peripheral immune system in patients with depression. Front Psychiatry (2017) 8:145 10.3389/fpsyt.2017.00145 28848457 PMC5553013

[2] Birur B Amrock EM Shelton RC Li L. Sex differences in the peripheral immune system in patients with depression. Front Psychiatry (2017) 8:108. 10.3389/fpsyt.2017.00108 28670290 PMC5472650

[3] Shelton RC Falola M Li L Zajecka J Fava M Papakostas GI. The pro-inflammatory profile of depressed patients is (partly) related to obesity. J Psychiatr Res (2015) 70:91–7. 10.1016/j.jpsychires.2015.09.001 26424427 PMC4734093

[4] Valkanova V Ebmeier KP Allan CL. CRP, IL-6 and depression: a systematic review and meta-analysis of longitudinal studies. J Affect Disord (2013) 150(3):736–44. 10.1016/j.jad.2013.06.004 23870425

[5] Hodes GE Kana V Menard C Merad M Russo SJ Neuroimmune mechanisms of depression. Nat Neurosci (2015) 18(10):1386–93. 10.1038/nn.4113 26404713 PMC4843114

[6] Cutolo M Seriolo B Villaggio B Pizzorni C Craviotto C Sulli A. Androgens and estrogens modulate the immune and inflammatory responses in rheumatoid arthritis. Ann N Y Acad Sci (2002) 966:131–42. 10.1111/j.1749-6632.2002.tb04210.x 12114267

[7] Angele MK Schwacha MG Ayala A Chaudry IH. Effect of gender and sex hormones on immune responses following shock. Shock (2000) 14(2):81–90. 10.1097/00024382-200014020-00001 10947147

[8] Endrighi R Hamer M Steptoe A. Post-menopausal women exhibit greater interleukin-6 responses to mental stress than older men. Ann Behav Med (2016) 50(4):564–71. 10.1007/s12160-016-9783-y 26943141 PMC4933724

[9] Li L Shelton RC Chassan RA Hammond JC Gower BA Garvey TW. Impact of major depressive disorder on prediabetes by impairing insulin sensitivity. J Diabetes Metab (2016) 7(4). 10.4172/2155-6156.1000664 27274905 PMC4892179

[10] Li L Garvey WT Gower BA. Childhood maltreatment is an independent risk factor for prediabetic disturbances in glucose regulation. Front Endocrinol (2017) 8:151. 10.3389/fendo.2017.00151 28713332 PMC5492465

[11] Mondelli V Vernon AC Turkheimer F Dazzan P Pariante CM. Brain microglia in psychiatric disorders. Lancet Psychiatry (2017) 4(7):563–72. 10.1016/s2215-0366(17)30101-3 28454915

[12] Meinlschmidt G Heim C. Decreased cortisol awakening response after early loss experience. Psychoneuroendocrinology (2005) 30(6):568–76. 10.1016/j.psyneuen.2005.01.006 15808926

[13] Holsboer F Von Bardeleben U Gerken A Stalla GK Muller OA Blunted corticotropin and normal cortisol response to human corticotropin-releasing factor in depression. N Engl J Med (1984) 311(17):1127 10.1056/nejm198410253111718 6090905

[14] Gold PW Chrousos G Kellner C Post R Roy A Augerinos P Psychiatric implications of basic and clinical studies with corticotropin-releasing factor. Am J Psychiatry (1984) 141(5):619–27. 10.1176/ajp.141.5.619 6324597

[15] Heim C Newport DJ Mletzko T Miller AH Nemeroff CB. The link between childhood trauma and depression: insights from HPA axis studies in humans. Psychoneuroendocrinology (2008) 33(6):693–710. 10.1016/j.psyneuen.2008.03.008 18602762

